# Renal Clearable H‐Dots Leveraging Ligand Complexation for Enhanced Active Tumor Targeting

**DOI:** 10.1002/smsc.202400246

**Published:** 2024-08-13

**Authors:** Yanan Cui, Seung Hun Park, Wesley R. Stiles, Atsushi Yamashita, Jason Dinh, Richard S. Kim, Yadong Zhang, Xiaoran Yin, Yoonji Baek, Haoran Wang, Kai Bao, Homan Kang, Hak Soo Choi

**Affiliations:** ^1^ Gordon Center for Medical Imaging Department of Radiology Massachusetts General Hospital and Harvard Medical School Boston MA 02114 USA; ^2^ School of Pharmacy Jining Medical College Rizhao Shandong 276826 China; ^3^ School of Pharmacy Shandong First Medical University & Shandong Academy of Medical Sciences Jinan Shandong 250021 China; ^4^ Department of Oncology The Second Affiliate Hospital of Xi'an Jiao Tong University Xi'an Shaanxi 710004 China

**Keywords:** active targeting, cyclic arginine–glycine–aspartic acids, nanoparticles, passive targeting, tumor targeting

## Abstract

The use of ligand conjugation onto nanoparticle surfaces as an active targeting strategy has gained significant attention in the pursuit of improving tumor‐specific delivery and retention. However, the chemical conjugation of targeting moieties often induces alterations in the physicochemical properties of nanoparticles, including size, conformation, charge‐to‐mass ratio, and hydrophilicity/lipophilicity, resulting in unexpected biodistribution and pharmacokinetic profiles. Here, the enhanced active targeting efficiency achieved by integrating cyclic arginine–glycine–aspartic acid (cRGD) peptides onto ultrasmall nanocarrier H‐dot while preserving its essential physicochemical and pharmacokinetic attributes is investigated. The resulting cRGD/H‐dots demonstrate improved cellular uptake via integrin α_v_β_3_ receptors, accompanied by negligible cytotoxicity. Notably, the active targeting efficacy of cRGD/H‐dots compared to unmodified H‐dots (1.2%ID/g, two‐fold increase) is quantitatively evaluated, validated through fluorescence imaging and histological analysis. The findings highlight that cRGD/H‐dots offer enhanced tumor targetability and prolonged tumoral retention while maintaining active renal clearance of unbound molecules.

## Graphical abstract

Renal clearable cyclic arginine–glycine–aspartic acid (cRGD)/H‐dots demonstrate enhanced active tumor targeting by forming complexes with cRGD, while maintaining essential characteristics including minimum nonspecific uptake and rapid urinary excretion. This study offers significant promise for cRGD/H‐dot as a prospective therapeutic nanoplatform in oncological interventions.

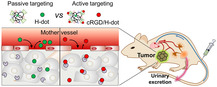

## 1 Introduction

**Figure 1:**
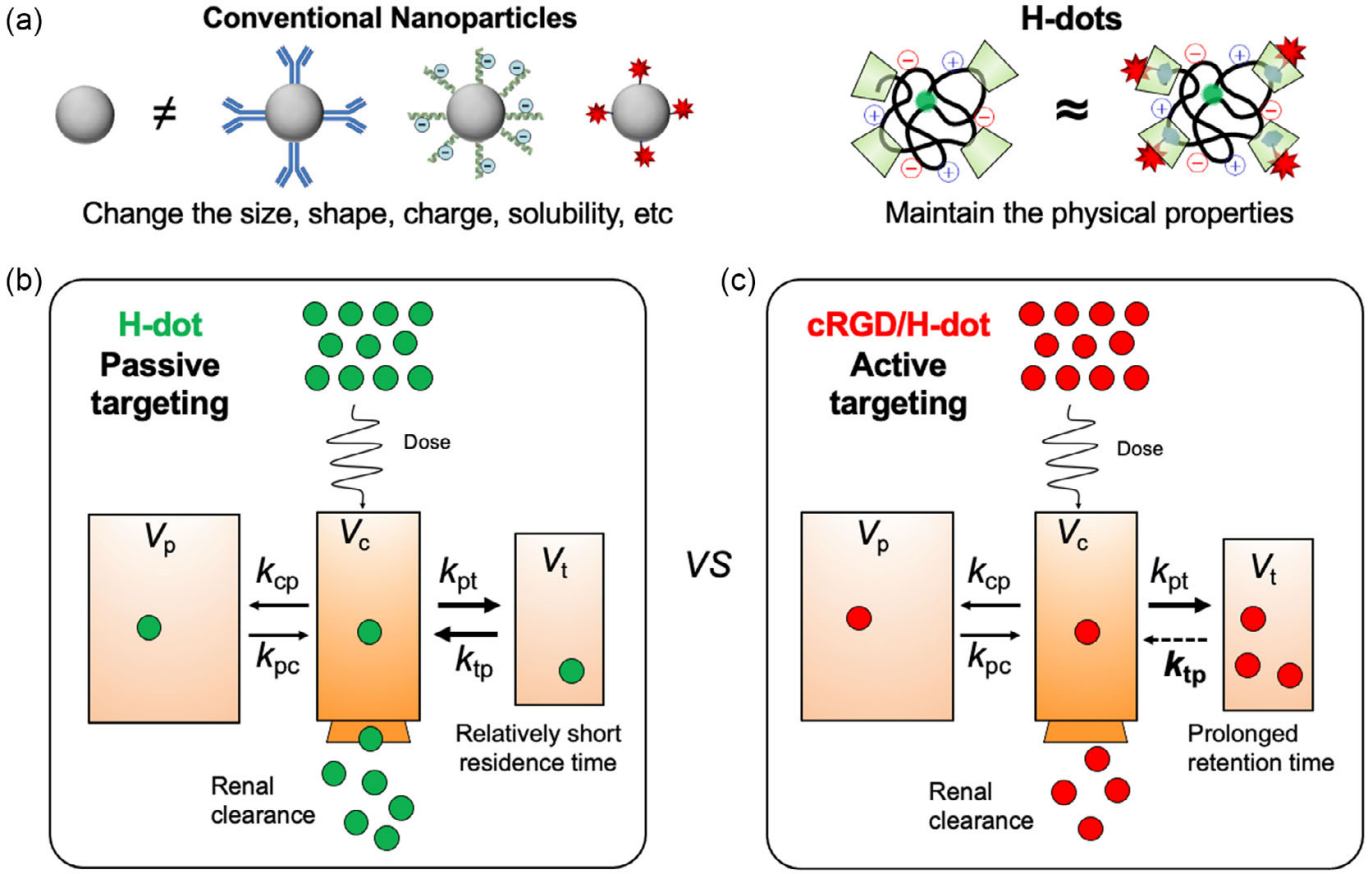
Passive versus active targeting. a) Schematic illustration of alterations postmodification of targeting moieties. cRGD/H‐dots retain their physicochemical properties following complexation with cRGD. b) Comparison of PK/dynamics, distribution, and clearance between H‐dot (passive targeting) and c) cRGD/H‐dot (active targeting). After administration, both H‐dot and cRGD/H‐dot accumulate at the tumor site via the EPR effect. However, cRGD/H‐dot can exhibit enhanced tumor‐targeting efficiency and prolonged retention compared to H‐dot, facilitated by α_v_β_3_ integrin targeting.

The efficiency of tumor targeting, measured as the percentage of injected dose (%ID), is of paramount importance in achieving significant therapeutic outcomes within nanoparticle (NP)‐based drug delivery systems and the broader field of nanomedicine. To this end, various targeting moieties, such as antibodies, peptides, DNA, ligands, etc., have garnered significant interest in improving interactions with the tumor cells and/or tumor microenvironment, the so‐called “active targeting strategy.”^[^ ^1^, ^2^, ^3^, ^4^, ^5^ ^]^ However, the introduction of targeting moieties onto the NP surface can induce notable alterations in the physicochemical properties of NPs, such as size (as indicated by relative molecular weight [MW] or hydrodynamic diameter, HD), morphology and conformation, charge distribution, and hydrophilicity/lipophilicity^[^ ^6^, ^7^, ^8^, ^9^, ^10^ ^]^ (**Figure** **1**a, left). These alterations result in different physiological behaviors, particularly in terms of transport efficiency to tumor sites,^[^ ^11^, ^12^, ^13^ ^]^ given that active targeting inevitably requires prior transportation of NPs to tumors facilitated by the enhanced permeability and retention (EPR) effect.^[^ ^14^, ^15^ ^]^ This poses difficulties in accurately comparing and evaluating the targeting efficacy of NPs with attached targeting moieties against unmodified NPs.^[^ ^4^, ^16^, ^17^ ^]^ For instance, while small NPs (5 nm) featuring targeting ligands exhibit a sixfold increase in %ID, their larger counterparts (30 nm) do not manifest significant enhancements in active targeting efficacy (merely 1.15‐fold higher).^[^ ^3^ ^]^ This incongruity may arise from the disproportionately greater impact of targeting ligands on smaller NPs. In the previous summary of more than 200 papers, active targeting strategy tended to outperform passive targeting approaches, resulting in delivery efficiencies of 0.9 and 0.6%ID, respectively; however, the enhancement was only 1.5‐fold.^[^ ^16^ ^]^

Previously, we reported an ultrasmall nanoplatform (<6.0 nm in HD), named Harvard‐dot (H‐dot), uniquely cleared through the kidney without significant nonspecific tissue uptake, showing promise in the gastrointestinal stromal tumor, lung cancer, and insulinoma treatments.^[^ ^18^, ^19^, ^20^, ^21^ ^]^ β‐cyclodextrin (β‐CD) on the H‐dot can form inclusion complexes with hydrophobic drugs due to van der Waals and hydrophobic interactions inside the cavity, and hydrogen bonding stabilizes the drug outside the cavity.^[^ ^18^ ^]^ The complexed drugs are typically stable in neutral physiological environments and efficiently release the drug in acidic tumor microenvironments (pH ≈ 6.0). Renal clearable H‐dots can significantly mitigate off‐target toxicity by fast clearance of off‐target drug/carrier to the bladder. In this study, we assessed the active targeting effect on the renal clearable H‐dot. The changes in size and charge distribution caused by introducing targeting moiety to H‐dots were minimized via the inclusion‐complex formation between targeting moiety and H‐dot via host–guest interactions (Figure 1a, right). As a result, the physicochemical properties and pharmacokinetic (PK) attributes remained consistent without notable alterations. Consequently, a valid comparison was drawn regarding the active targeting efficacy within the similar PK profiles between H‐dot and cRGD‐complexed H‐dots (cRGD/H‐dot; Figure 1b). Our research shows that the active tumor‐targeting strategy contributes to advancing NP‐based drug delivery systems for precise and effective treatment.

## 2 Results

### 2.1 Preparation and Characterization of cRGD‐Loaded H‐dots (cRGD/H‐dots)

**Figure 2:**
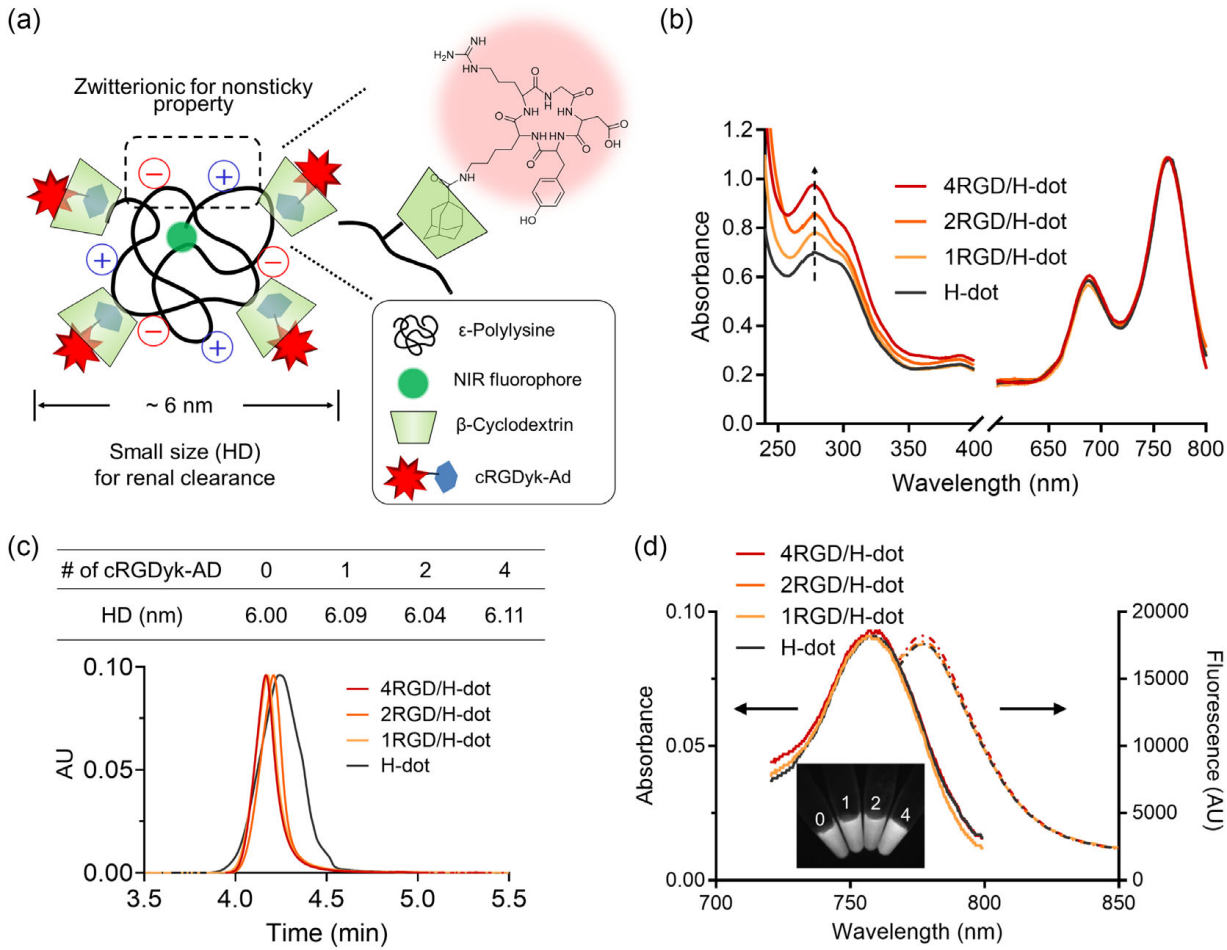
Physicochemical characteristics of cRGD/H‐dots. a) Schematic depiction of cRGD/H‐dot formation. b) Absorption spectra of cRGD/H‐dots with various numbers of cRGDs into H‐dot. c) Size exclusion column chromatography result for H‐dot and cRGD/H‐dots and calculated HD. d) Absorbance (solid lines) and fluorescence (dashed lines) spectra of H‐dot and cRGD/H‐dots in saline (50 μM).

H‐dot comprises zwitterionic ε‐poly‐L‐lysine (EPL), β‐CD, and a near‐infrared (NIR) fluorophore for biodistribution, hydrophobic drug encapsulation, and imaging, respectively (**Figure** **2**a). H‐dot was prepared by a previously reported synthetic route (Figure S1, Supporting Information). The average number of β‐CDs on the EPL backbone was calculated to be 7.4 by ^1^H‐NMR analysis (Figure S2, Supporting Information). The zwitterionic surface was reflected to be 50% succinylation ratio and confirmed by ninhydrin assay (Figure S3, Supporting Information). We employed cRGD peptides to target integrin α_v_β_3_ and α_v_β_5_, overexpressed on various cancer cell surfaces.^[^ ^22^, ^23^, ^24^, ^25^ ^]^ The cyclic structure of cRGD confers robust stability compared to linear peptides, offering resilience against enzymatic degradation.^[^ ^26^ ^]^ This stability is pivotal for sustaining the ligand's integrity throughout its journey in the body, thereby extending its circulation time and bolstering its efficacy in reaching and engaging with tumor sites.

To complex cRGDyK into the hydrophobic cavity of β‐CD through host–guest interactions, adamantane (Ad) was conjugated with the amine of lysine on cRGDyK. Ad is well known to form a strong inclusion complex with β‐CD (*K*a = 2.8 × 10^4^ M^−1^),^[^ ^27^ ^]^ leading to the stability of H‐dots during systemic circulation and tumor targeting. The purity of cRGD‐Ad was found to be 95.28% by high perfomance liquid chromatography‐mass spectrometry (HPLC‐MS) (retention time ≈8.1 min, MW 782 Da) (Figure S4, Supporting Information). Different equivalents of cRGD‐Ad (2, 4, and 8) were mixed with H‐dots to prepare cRGD/H‐dots with various numbers of cRGD‐Ad. The number of cRGDs on H‐dots was determined using UV–vis absorbance and ^1^H‐NMR spectroscopy (Figure 2b, S2, and S5, Supporting Information). The absorbance of cRGD‐Ad at 276 nm in the UV–vis spectrum, alongside the proton peaks observed at 6.8 and 7.2 ppm in the ^1^H‐NMR spectra, exhibited a direct correlation with the increasing equivalents of cRGD‐Ad. Interestingly, adding 2, 4, and 8 equivalents of cRGD‐Ad resulted in 1, 2, and 4 cRGD‐Ad complexations on an H‐dot, respectively. This result suggests that inclusion complex formation was precisely controlled over the ratio between cRGD‐Ad and β‐CD. We denoted as nRGD/H‐dots, where ‘n’ represents the number of cRGDs.

Next, we assessed the minimal impact of cRGD‐Ad quantities on the properties of cRGD/H‐dots, focusing on the HD and fluorescence signal changes of cRGD/H‐dots. Intriguingly, the HDs of both H‐dot and cRGD/H‐dots were consistently measured to be ≈6 nm, even in the case of 4RGD/H‐dot (deviation of HD < 2%) (Figure 2c, S6, Supporting Information), indicating that the alteration on tumor targetability due to size‐dependent EPR effect will be minimized. In addition, the HD of cRGD/H‐dots remained smaller than the glomerular filtration threshold after loading cRGD‐Ad.^[^ ^28^ ^]^ The variations in absorbance and fluorescence intensity between H‐dot and cRGD/H‐dot solutions (50 μM) were marginal, measuring less than 1.1% and 1.7%, respectively (Figure 2d). Furthermore, the minimal disparities in fluorescent signals between H‐dot and cRGD/H‐dots were observed using our fluorescence imaging system (FIAT‐L).

To assess the effect of modification of cRGD‐Ad on the surface charge of RGD/H‐dot, we conducted a stability test of H‐dot and RGD/H‐dot in various solutions such as phosphate buffer saline (PBS) (pH 5, 6, and 7) and PBS (pH 5, 6, and 7) supplemented with 10% fetal bovine serum. Measurements for H‐dot and 4RGD/H‐dot did not show any significant decrease in absorbance across all conditions over a 48 h period (Figure S7a–c, Supporting Information), indicating no aggregation of H‐dot and 4RGD/H‐dot. Furthermore, the results suggest that the incorporation of cRGD‐Ad does not significantly alter the surface charge of RGD/H‐dots after the complexation of 4RGD onto H‐dots. These results indicate the successful integration of cRGD‐Ad into H‐dots, with minimal impact on their inherent physicochemical properties.

### 2.2 In Vitro Cell Binding Affinity and Cytotoxicity of cRGD/H‐dot

To access the binding affinity of cRGD/H‐dot to integrin α_v_β_3_, Lewis lung carcinoma (LLC) and human nonsmall cell lung carcinoma (H23) cell lines were chosen due to their differing expression levels of integrin α_v_β_3_.^[^ ^29^, ^30^ ^]^ LLC and H23 cell lines were treated with 4RGD/H‐dot (5 μM) and incubated for 2 h prior to imaging (Figure S8, Supporting Information). Fluorescence microscopic imaging exhibited negligible uptake of H‐dot and 4RGD/H‐dot by both cell lines within the initial 30 min postincubation. 4RGD/H‐dot, however, showed considerable intracellular uptake compared to H‐dot in LLC cells 2 h postincubation (****p < 0.0001) while both H‐dot and 4RGD/H‐dot showed negligible intracellular uptake in integrin α_v_β_3_ negative H23 cells (****p < 0.0001 for 4RGD/H‐dot). The enhanced cellular uptake of cRGD/H‐dot in LLC cells is attributed to the active targeting of cRGD on the overexpressed integrins in LLC cells.^[^ ^31^ ^]^

The cytotoxicity of H‐dot and cRGD/H‐dot on NIH3T3 fibroblast cells was conducted. Concentrations ranging from 5 to 100 μM were administered to the cells, and their viability was evaluated using the Cell Counting Kit‐8 (CCK‐8) assay. Notably, both cRGD/H‐dot and H‐dot showed no significant impact on the viability of NIH3T3 cells within the concentration up to 100 μM, as shown in Figure S9, Supporting Information. Overall, these results suggest that cRGD/H‐dots possess the ability to selectively target cancer cells that overexpress α_v_β_3_ receptors while sparing normal cells from cytotoxic effects.

### 2.3 In Vivo Biodistribution and Pharmacokinetics of cRGD/H‐dots

**Figure 3:**
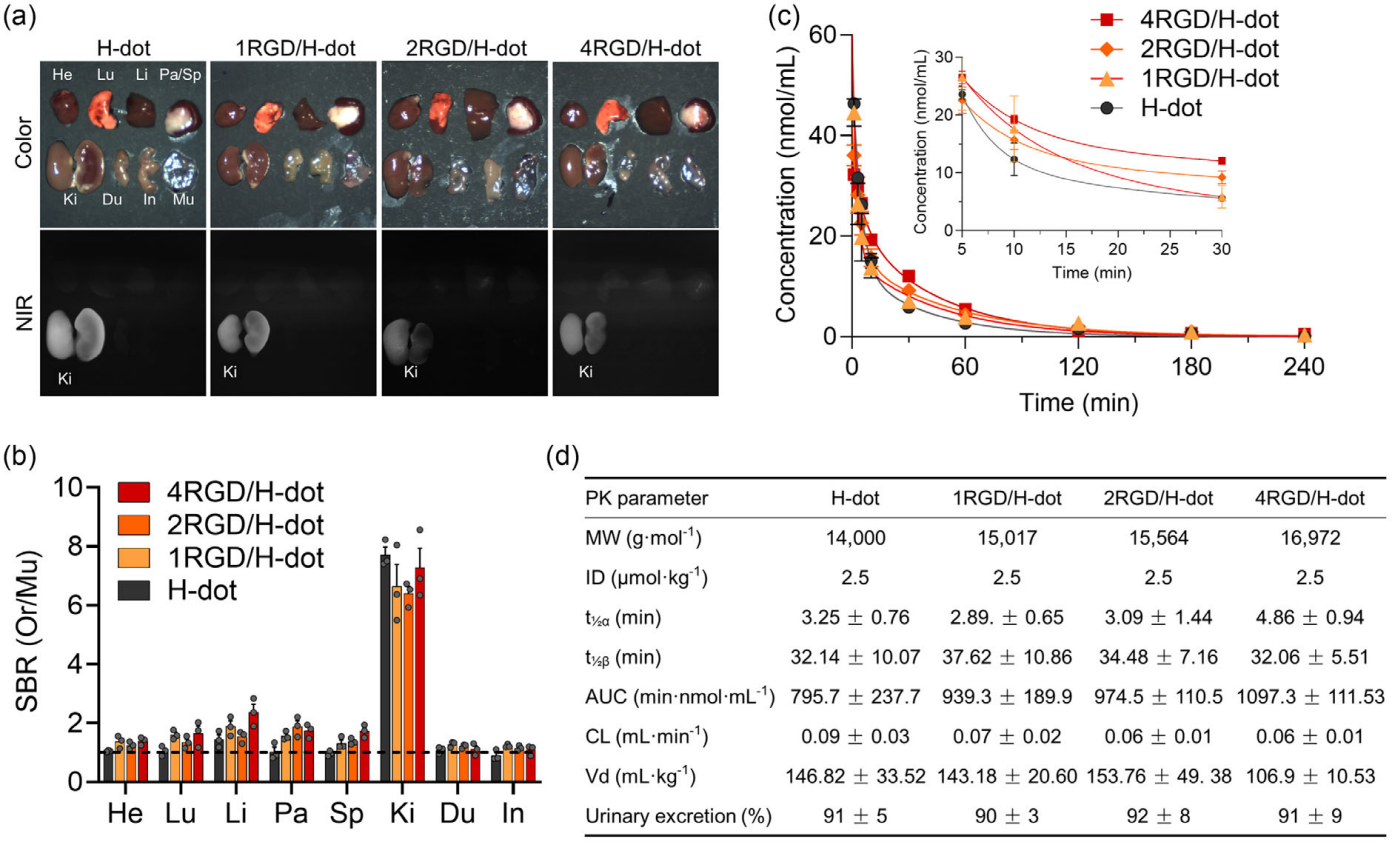
In vivo behavior of cRGD/H‐dot. a) Biodistribution analysis of H‐dot and cRGD/H‐dots at 4 h post‐injection. b) SBR of resected organs from mice administered with H‐dot or cRGD/H‐dots (the dashed line indicates 1, *n* = 3 per group, mean ± s.e.m.). c) Plasma concentration decay curves of H‐dot and cRGD/H‐dots (*n* = 3 per group, mean ± s.e.m.). d) PK parameters comparison between H‐dot and cRGD/H‐dots.

Before conducting in vivo tumor‐targeting studies, the biodistribution and PK of cRGD/H‐dot complexes were evaluated. CD‐1 mice were injected with free H‐dots or cRGD/H‐dots, and the major tissues and organs were resected for observation under the NIR imaging 4 h postinjection. The NIR fluorescence signals from cRGD/H‐dots were primarily localized within the urinary system (kidneys and bladder) with no statistical significance compared to H‐dot alone (**Figure** **3**a,b). In terms of PK analysis, plasma concentration decay curves were generated using fluorescence signals of H‐dots collected at predetermined time points (Figure 3c and S10, Supporting Information), and PK parameters are summarized in Figure 3d. The distribution (*t*_1/2α_) and elimination (*t*_1/2β_) half‐lives for all cRGD/H‐dots did not significantly deviate from those of H‐dot alone, demonstrating 3 and 30 min, respectively. This result indicates that all H‐dot complexes show rapid systemic distribution and rapid elimination from the bloodstream.^[^ ^32^, ^33^ ^]^ In addition, no significant differences were observed in the other PK parameters, suggesting that H‐dots are not affected by cRGD complexation with no evident changes in biodistribution, PK properties, and renal clearance (>90%ID, 4 h postinjection).

### 2.4 In Vivo Tumor‐Targeting Efficiency of cRGD/H‐dots

**Figure 4:**
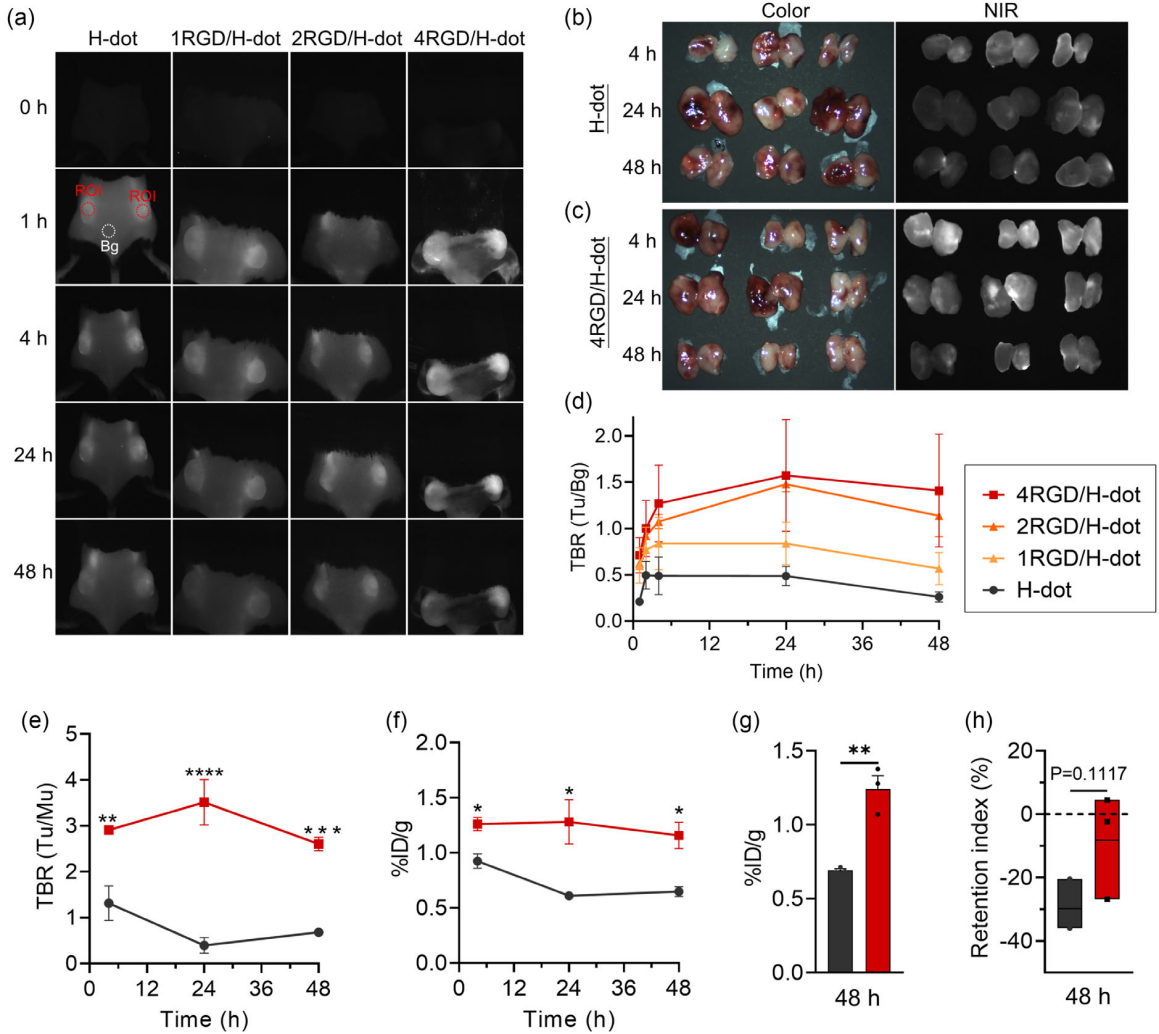
Tumor‐targeting efficacy of cRGD/H‐dots. a) NIR fluorescence imaging until 48 h postinjection of H‐dot and cRGD/H‐dots. b) Color and NIR fluorescence images of dissected tumors from mice injected with H‐dot and c) 4RGD/H‐dot. d) TBR of tumor sites from mice injected with cRGD/H‐dots compared to H‐dot. e) TBR from dissected tumors 4RGD/H‐dots compared to H‐dot. f) Delivery efficiency (% injection dose/gram, %ID/g), g) mean values of delivery efficiency until 48 h, and h) RI of H‐dot and 4RGD/H‐dot. *n* = 3 per group, mean ± s.e.m., *p* values <0.05 were considered significant: *p < 0.05, **p < 0.01, ***p < 0.001, ****p < 0.0001.

LLC cells were inoculated subcutaneously into C57BL/6 mice to establish a lung cancer mouse model. Following this, the tumor‐bearing mice were administered with 1, 2, and 4RGD/H‐dots. The fluorescence signals in the tumor region were monitored for 48 h postinjection using FIAT‐L, and the tumor‐to‐background ratio (TBR) was calculated (**Figure** **4**a–e, S11, Supporting Information). Particularly, the TBR of 4RGD/H‐dots and 2RGD/H‐dots groups increased over time up to 24 h postinjection, followed by showing a slight decline thereafter (Figure 4d). Conversely, H‐dot alone and 1RGD/H‐dot groups showed diminishing TBRs after 4 h. For meticulous comparison, 4RGD/H‐dots were selected and specifically compared with free H‐dots. As shown in Figure 4e, 4RGD/H‐dots demonstrated ≈3‐fold higher TBRs compared to H‐dots at all time points (**p < 0.005). Quantitative analysis demonstrated that the percentage of ID per gram of tumor (%ID/g) in the 4RGD/H‐dot group showed significantly higher values in all time points (*p < 0.05), and the mean value was ≈1.2%ID/g, which is twofold higher compared to H‐dot alone (0.6%ID/g) (Figure 4f,g). Furthermore, the retention index (RI) of 4RGD/H‐dot (−8.2) reveals a noticeable trend toward prolonged retention within the tumor, attributed to the active targeting facilitated by cRGD complexation (Figure 4h). This enhancement in delivery efficacy is observed in comparison to that of H‐dot alone (−29.8).^[^ ^19^, ^20^ ^]^ These findings suggest that the significant enhancement in tumor‐targeting efficiency and prolonged retention was achieved through the incorporation of cRGD into H‐dot, leveraging a combination of passive and active targeting mechanisms.

### 2.5 Histological Analysis of Tumors with H‐dot or 4RGD/H‐dot

**Figure 5:**
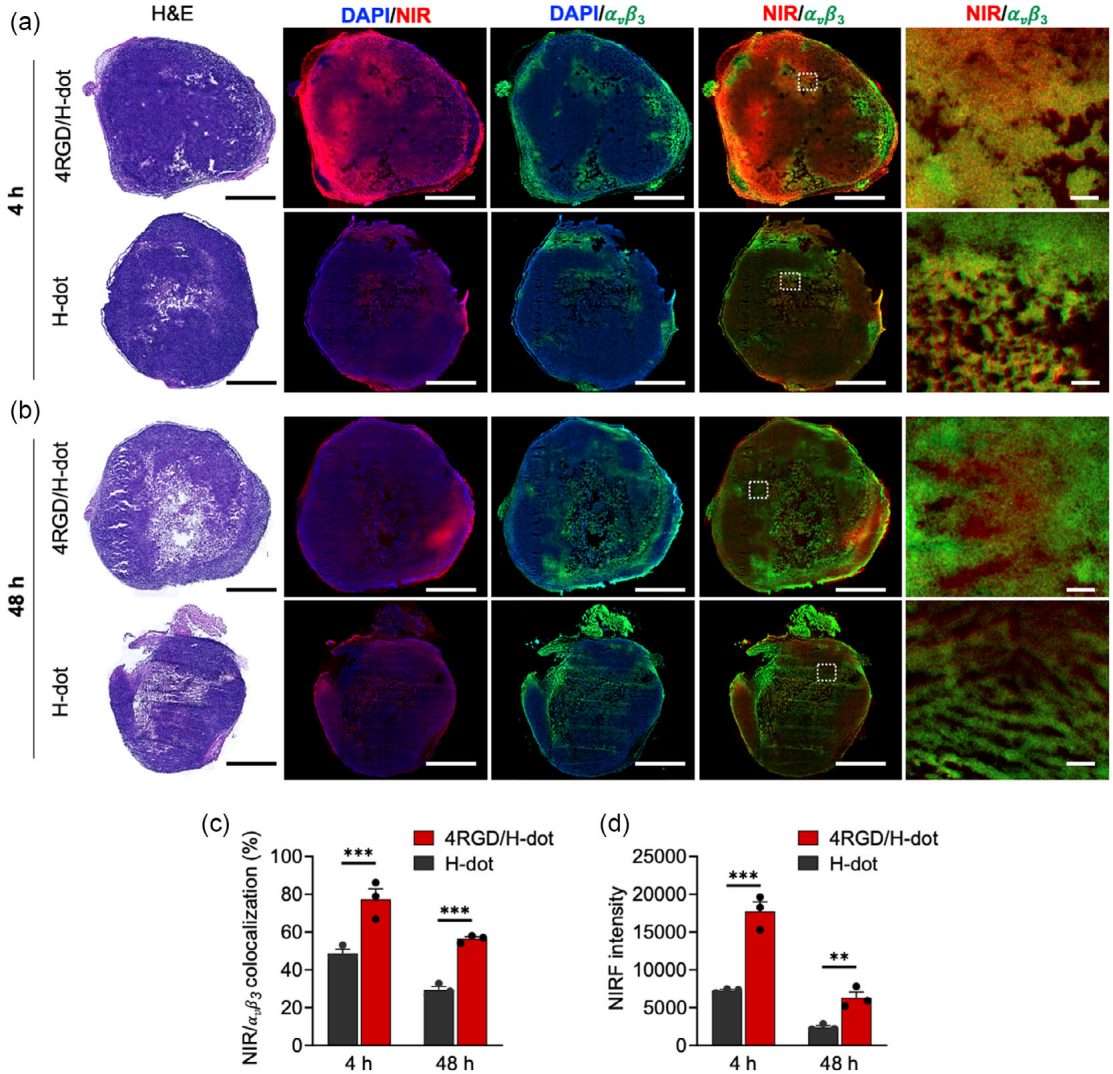
Histological analysis of dissected tumors for targeting efficacy of H‐dot and 4RGD/H‐dot. a) H&E staining images and fluorescence images of immunofluorescence staining for integrin α_v_β_3_ (green) and NIR fluorescence (red) for dissected tumors injected with H‐dot and 4RGD/H‐dot at 4 and b) 48 h postinjection. Scale bar: 1 mm and 50 μm in whole and magnified images, respectively. c) The ratio of colocalization area between NIR and α_v_β_3_, and d) NIR fluorescence intensity of H‐dot and 4RGD/H‐dot at each time point. *p* values <0.05 were considered significant: **p < 0.01, ***p < 0.001.

Histological analysis was followed to elucidate whether the enhanced targeting efficiency of cRGD/H‐dots resulted from the specific interaction between cRGD and integrin α_v_β_3_ on tumor cells (**Figure** **5**). Hematoxylin and eosin (H&E) staining was employed to confirm tumor morphology, and immunofluorescence staining was utilized to verify α_v_β_3_ expression (green). At 4 h post‐injection, both H‐dot and 4RGD/H‐dot exhibited deep tumor penetration, consistent with previous findings.^[^ ^19^, ^20^ ^]^ However, notably higher NIR fluorescence (red) was observed in the 4RGD/H‐dot group compared to H‐dot alone, indicating increased accumulation of 4RGD/H‐dot. Moreover, the merged images combining NIR and α_v_β_3_ signals exhibited a significant colocalization between α_v_β_3_ expression and NIR fluorescence in 4RGD/H‐dot, compared to that in the free H‐dot group (***p < 0.001; Figure 5a). This result indicates that 4RGD/H‐dots benefited from both passive and active targeting mechanisms with improved tumor localization and retention while H‐dots relied primarily on passive targeting via the small‐sized EPR effect.^[^ ^10^ ^]^ Surprisingly, NIR signals in the 4RGD/H‐dot group at 48 h postinjection still remained higher compared to free H‐dots (**p < 0.01), and substantial colocalization with α_v_β_3_ was observed in several other tissue components (Figure 5b). However, NIR signals in both groups notably diminished compared to at 4 h, suggesting that H‐dots diffused into the tumoral tissue are eventually cleared out after delivering drugs to the target tissue. These results imply that cRGD/H‐dot as a drug delivery nanoplatform holds promise as an effective vehicle for drug delivery and unbound molecules clear from normal tissues, thereby minimizing potential side effects associated with the accumulation of drug delivery vehicles.

## 3 Discussion

NPs have emerged as promising carriers for targeted tumor therapy, offering potential advantages over traditional therapeutics.^[^ ^34^ ^]^ The EPR effect has long been considered the cornerstone of tumor targeting in nanomedicine.^[^ ^35^ ^]^ Although EPR‐mediated passive targeting has demonstrated efficacy, challenges persist in attaining optimal tumor specificity and therapeutic outcomes.^[^ ^10^ ^]^ These limitations of the EPR effect have prompted considerable interest in active targeting strategies involving the attachment of targeting moieties to NP surface to enhance tumor targeting and retention.^[^ ^5^, ^36^, ^37^ ^]^

However, questions have been raised regarding the superior efficacy of active targeting compared to passive targeting, particularly considering that the improvement in delivery efficiency has been marginal.^[^ ^16^ ^]^ To accurately assess the active targeting efficiency of NPs, it is crucial to maintain consistency in key characteristics influencing the EPR effect, such as size, shape, charge, conformation, and hydrophilicity/lipophilicity, across unmodified NPs.^[^ ^6^ ^]^ This ensures that any differences in targeting efficacy can be attributed solely to the active targeting moiety rather than variations in EPR‐related properties. Previous efforts have often involved extensive modifications of NPs to introduce active targeting moieties, inadvertently altering their physicochemical properties, thus complicating the assessment of the active targeting effect. To address these challenges, our study aimed to assess the active targeting effect of renal clearable H‐dots via complexation of active ligand cRGD, thereby achieving the following meaningful results.

First, we minimized the changes in the physicochemical and biodistribution/PK properties of H‐dots postcomplexation with cRGD‐Ad. Potential challenges that arise with the integration of antibodies or peptides are increasing the overall HD and surface properties of native NPs due to their relatively large MW. Additionally, introducing DNA‐targeting moieties may alter the surface charge of NPs, typically resulting in negative charges.^[^ ^38^ ^]^ To overcome these challenges, cRGD was loaded onto the H‐dot surface using host–guest interactions between Ad and β‐CD. This approach ensures that cRGD/H‐dots maintain the physicochemical properties of native H‐dots while effectively incorporating targeting moieties.

Second, cRGD/H‐dots are capable of rapid clearance without nonspecific binding, attributed to their zwitterionic surface and small HD.^[^ ^18^ ^]^ To mitigate off‐target accumulation, we aimed to preserve these inherent advantages of H‐dots even after integrating cRGD. Our investigations confirmed that cRGD/H‐dots maintain the original biodistribution and PK properties of unmodified H‐dots, eventually excreting them to the urinary bladder. This characteristic not only expedites systemic clearance but also mitigates the potential risk of off‐target toxicity.

Lastly, the active targeting effect of cRGD/H‐dots was quantitatively compared with the passive targeting achieved by the EPR effect of free H‐dots. Given that cRGD/H‐dots retain the physical and PK properties of H‐dots, the enhanced targeting efficiency observed in the targeting experiment was attributed to the active effect of cRGD rather than accumulation via the EPR effect or alterations in other physicochemical properties. This highlights the specific targeting effect achieved by the incorporated cRGD. Moreover, considering that cRGD/H‐dots exhibit a higher degree of colocalization with α_v_β_3_ compared to H‐dots, they are anticipated to be more efficacious when employed in tumors expressing high levels of α_v_β_3_ than LLC tumor models utilized in this experiment.^[^ ^39^ ^]^

Although this study shows promising results, its interpretations are circumscribed by specific constraints. While we examined cRGD/H‐dots containing up to 4 cRGD, it is uncertain how well this applies to cRGD/H‐dots with more cRGD. Given that H‐dot holds ≈8 β‐CDs, it has the potential capacity to host up to eight cRGD moieties. However, only a maximum of four cRGDs were complexed into a single H‐dot (≈50% loading efficiency), likely due to the steric hindrance and restricted molecular conformation. Additionally, our study mainly used LLC tumors as a model, which might not cover all types of cancers with varying levels of α_v_β_3_ expression. Extending our investigations to include other cancer models with different α_v_β_3_ expression profiles could provide valuable insights into the targeting efficacy of cRGD/H‐dots across diverse tumor types and tumor microenvironments.

In summary, the evaluation of the cRGD/H‐dot's active targeting efficacy was undertaken through its complexation with cRGD while retaining key attributes such as minimum nonspecific uptake and rapid urinary excretion. Our results emphasize its considerable potential as a promising therapeutic nanoplatform in oncological interventions. The refinement of active targeting methodologies, elucidation of mechanisms, and sustained exploration of translation to clinical application hold the prospect of advancing cancer theragnosis in the nanomedicine field.

## 4 Experimental Section

4.1

4.1.1

##### Preparation of Adamantane‐Conjugated CRGDyK (cRGD‐Ad)

To prepare cRGD‐Ad, 100 mg of cRGDyK was dispersed in 50 mL of MeOH. Subsequently, 160 μL of *N*,*N*‐diisopropylethylamine (DIEA) was added to the cRGDyK solution, and the pH was adjusted to ≈8.5–9.0. Next, 48 mg of 1‐adamantanecarboxyl chloride was added to the mixture, and the reaction proceeded at room temperature overnight. After completion of the reaction, the reaction mixture was precipitated into 500 mL of ethyl acetate, followed by centrifugation at 3500 rpm for 15 min at 4 °C.

##### Preparation and Characterization of cRGD‐Ad‐Loaded H‐Dot Complexes (cRGD/H‐dots)

To prepare cRGD/H‐dot complexes, cRGD‐Ad was dissolved in DMSO at a concentration of 200 mM. H‐dot powder was dissolved in DI water at a concentration of 1 mM. Subsequently, 11, 22, and 44 μL (2, 4, and 8 equivalent, respectively) of cRGD‐Ad solutions were added to 1 mL of H‐dots to achieve complexes with cRGD‐Ad to achieve H‐dot molar ratios of 1, 2, and 4, respectively. The mixtures were vortexed for 120 min at room temperature and centrifuged at 14 000 rcf for 10 min to precipitate impurities. The supernatants were purified using spin columns with a MW cutoff of 10 k (centrifugation at 12 000 RCF for 20 min) and freeze dried. The molar ratio of cRGD‐Ad to H‐dot was determined using UV spectrophotometry and ^1^H‐NMR spectroscopy.

##### In Vitro Cellular Uptake of cRGD/H‐dot

To confirm the cellular uptake of cRGD/H‐dot by integrin α_v_β_3_ overexpressed tumor cells, LLC and H23 cells were seeded at a density of 2 × 10^4^ cells per well in 96‐well plates and incubated for 24 h. After the incubation period, the cells were washed with 500 μL of PBS. Subsequently, cRGD/H‐dot complexes or H‐dots were dissolved in fresh PBS at a concentration of 5 μM. Following the addition of cRGD/H‐dot complexes or H‐dot solutions to the cells according to the experimental groups, the cells were further incubated for specified durations: 30 min and 2 h, respectively. At each time point, the cells were washed three times with 500 μL of PBS to remove any residual H‐dots. Subsequently, the cells were fixed with 500 μL of 4% paraformaldehyde for 20 min. After fixation, the cells were washed thrice with 500 μL of PBS. Finally, the cells were observed using Cytation5 (BioTek Instruments) for analysis of cellular uptake and localization of H‐dots. All images of each group were taken at randomly chosen locations in the well plate. All cells in the bright‐field images were counted, and then NIR‐positive cells were counted in the merged bright‐field and NIR fluorescence images. The following equation was used to calculate the NIR‐positive cell ratio.

(1) $$\text{NIR positive cell ratio }\left(\%\right)=\frac{\text{Number of NIR positve cells}}{\text{Number of total cells}}\times 100$$

##### In Vitro Cytotoxicity of cRGD/H‐dots

For the cytotoxicity assessment, NIH3T3 cells were treated with varying concentrations of cRGD/H‐dots ranging from 1 to 100 μM. After 24 h of treatment, NIH3T3 cells were washed twice with PBS. Subsequently, 100 μL of fresh complete medium was added to each well, followed by the addition of 10 μL of CCK8 solution to each well. After incubating for 4 h, absorbance spectra were measured at 450 nm using a microplate reader (Cytation5). All experiments were performed with three replicates to ensure statistical reliability.

##### In Vivo Biodistribution and Pharmacokinetics of cRGD/H‐dot Complexes

Animals were housed in an AAALAC‐certified facility and were studied under the supervision of the MGH IACUC in accordance with the approved institutional protocol (# 2016N000136). Prior to treatment injection, male CD‐1 mice aged 6 weeks and weighing 25–30 g were anesthetized with isoflurane and oxygen. Blood samples were collected at the 0 min time point by slightly cutting the end of the tail and collecting it in capillary tubes (Fisher Scientific, Pittsburgh, PA). Intravenous injections of H‐dot and cRGD/H‐dot complexes in saline were administered at 2500 nmol kg^−1^. Blood samples were drawn at various time points postinjection (1, 3, 5, 10, 30, 60, 120, 180, and 240 min). The fluorescence intensities of serum samples in capillary tubes were measured to determine distribution (*t*_1/2α_) and elimination (*t*_1/2β_) half‐life values (*n* = 3–4 for each group). After 4 h post‐injection, mice were euthanized for organ imaging, including the liver, lung, spleen, kidney, stomach, intestine, and bladder. The fluorescence intensities of serum and organs were assessed using the FIAT‐L system.

##### In Vivo Targeting Efficacy of cRGD/H‐dot Complexes

To establish the LLC tumor model, C57BL/6 mice were subcutaneously injected with 2 × 10^5^ LLC cells suspended in 100 μL of DMEM/Matrigel (50 v/v%) in the flank region. Real‐time imaging was conducted once tumors had formed after 2 weeks. LLC lung tumor‐bearing mice were then intravenously administered H‐dot or cRGD/H‐dots at the same dose used in the biodistribution and PK experiments. In vivo fluorescence imaging was performed using the FIAT‐L system for 48 h postinjection. Subsequently, the mice were euthanized for ex vivo imaging, %ID/g was calculated, and histological evaluations of various organs were done, including the heart, liver, stomach, lung, kidney, spleen, intestine, bladder, pancreas, muscle, and tumor.

##### Delivery Efficiency of H‐dot and cRGD/H‐dot

To calculate the delivery efficiency of H‐dot and cRGD/H‐dots over time, the linear trapezoidal method was used to calculate the area‐under‐the‐curve (AUC_Tumor_) of the plot of %ID/g in the tumor as a function of time. The following equations were used to calculate the mean value of delivery efficiency.

(2) $$\text{Trapezoid }\left(T_{i}\;\right)=\;0.5\;\left(C_{i}\;+\;C_{i-1}\right)\left(t_{i}\;–\;t_{i-1}\right)$$

(3) $${\text{AUC}}_{\text{Tumor}}=\sum_{i=1}^{n}T_{i}\;$$

(4) $$\text{Delivery efficiency}=\;\frac{{\text{AUC}}_{\text{Tumor}}}{t_{\text{end}}-t_{1}}$$

where *C*_*i*_ is %ID/g at the dissected time (*t*_*i*_). The AUC_Tumor_ is calculated by summation of all *T*_*i*_ from *i* = 1 to *i* = *n*.

##### Retention Index (RI) of H‐dot and cRGD/H‐dot

To calculate the RI of H‐dot and cRGD/H‐dots, the following equation was used.

(5) $$\text{RI }\left(\%\right)=\left[\left({\text{DE}}_{48h}-{\text{DE}}_{4h}\right)/{\text{DE}}_{4h}\right]\times 100$$

where DE_t_ is %ID/g at the denoted time *t*. For %ID/g_4h_, mean values of %ID/g of H‐dot or cRGD/H‐dot at 4 h were used for fair calculation.

##### Retention Index (RI) of H‐dot and cRGD/H‐dot: Tumor Histopathology Evaluation

The tumor tissues were embedded in the Tissue‐Tek optimum cutting temperature compound (Sakura Finetek, Torrance, CA) without a prefixation step. The tissue block was frozen at −80 °C. Frozen sections were cut at a thickness of 10 or 20 μm by a cryostat (Leica, Germany). Then, the tissue sections were stained with H&E. The expression of integrin α_v_β_3_ was evaluated by staining with the respective antibodies, and then the stained tumor tissues were counterstained with DAPI. We used the BioTek Cytation 5 (Winooski, VT) for pathological fluorescence imaging and observation.

##### Retention Index (RI) of H‐dot and cRGD/H‐dot: Image and Statistical Analyses

Fluorescence and background intensities in specific tissue regions were quantified utilizing customized imaging software and ImageJ v1.48 (National Institutes of Health, Bethesda, MD). The signal‐to‐background ratio (SBR) was computed as SBR = fluorescence/background, where the background denotes the fluorescence intensity of muscle tissue. Data were presented as mean ± standard error of the mean (s.e.m.) from at least three biological replicates. The students’ t‐test was employed for statistical analysis to assess the significance of the results. Moreover, differences among groups were evaluated using one‐way analysis of variance to determine statistical disparities among more than two groups. A p‐value below 0.05 was considered statistically significant, with significance levels annotated as follows: *p < 0.05, **p < 0.01, ***p < 0.001, and ****p < 0.0001.

## Conflict of Interest

The authors declare no conflict of interest.

## Author Contributions

**Yanan Cui**, **Seung Hun Park**, **Homan Kang**, and **Hak Soo Choi** conceived the study and contrived the experiments. **Yanan Cui**, **Seung Hun Park**, **Wesley R. Stiles**, **Haoran Wang**, **Kai Bao**, **Richard S. Kim**, and **Yadong Zhang** synthesized and characterized H‐dots. **Yanan Cui**, **Seung Hun Park**, **Atsushi Yamashita**, **Jason Dinh**, **Xiaoran Yin**, and **Yoonji Baek** performed the biodistribution and pharmacokinetic experiments. All authors analyzed and interpreted the data. **Yanan Cui**, **Seung Hun Park**, and **Wesley R. Stiles** wrote the initial manuscript draft. **Seung Hun Park**, **Homan Kang**, and **Hak Soo Choi** edited the manuscript. **Homan Kang** and **Hak Soo Choi** supervised the entire project. All authors contributed to revising the manuscript. All authors have approved the final version of the manuscript.

## Supporting information

Supplementary Material

## Data Availability

The data that support the findings of this study are available in the supplementary material of this article.
