# Supplementary material for: Renal Clearable H‐Dots Leveraging Ligand Complexation for Enhanced Active Tumor Targeting
Source: Small Sci. 2024 Aug 13;4(11):2400246. doi: 10.1002/smsc.202400246 (PMC11935198; doi:10.1002/smsc.202400246)
Supplement: Supplementary file 1 — Supplementary Material [file SMSC-4-2400246-s001.pdf]

Supporting Information

Renal Clearable H-dots Leveraging Ligand Complexation for Enhanced Active Tumor Targeting

Yanan Cui,[1,2,#] Seung Hun Park,[1,#] Wesley R. Stiles,[1] Atsushi Yamashita,[1] Jason Dihn,[1] Richard S. Kim,[1] Yadong Zhang[3], Xiaoran Yin,[1,4] Yoonji Baek,[1] Haoran Wang,[1] Kai Bao,[1] Homan Kang,[1,]* and Hak Soo Choi[2,]*

[1]Gordon Center for Medical Imaging, Department of Radiology, Massachusetts General Hospital and Harvard Medical School, Boston, MA 02114, USA

[2]School of Pharmacy, Jining Medical College, Rizhao, Shandong, 276826, China

[3]School of Pharmacy, Shandong First Medical University & Shandong Academy of Medical Sciences, Jinan, Shandong, 250021, China

[4]Department of Oncology, The Second Affiliate Hospital of Xi'an Jiao tong University, Xi'an, Shaanxi, 710004, China

#These authors contributed equally to this work

The file includes:

Supplementary Methods

Figure S1. Synthetic procedure for H-dot

Figure S2. ^{1}H-NMR chromatography of H-dot and RGD/H-dot complexes

Figure S3. Ninhydrin assay of the amine residue on H-dots

Figure S4. Synthesis of cRGD-Ad

Figure S5. Absorption spectra of cRGD-Ad

Figure S6. SEC-HPLC spectra for H-dot and RGD/H-dot

Figure S7. Stability of H-dot and RGD/H-dot

Figure S8. In vitro cellular binding tests for H-dot and RGD/H-dot

Figure S9. Cell viability of NIH3T3 cell of H-dot and RGD/H-dot

Figure S10. Blood samples of H-dot and 4RGD/H-dot in capillary tubes

Figure S11. Biodistribution of H-dot and 4RGD/H-dot

SUPPLEMENTARY METHODS

Reagents and materials: Epsilon-Polylysine (ε-poly-L-lysine, EPL; MW ~3,900) was purchased from BOC Sciences (Shirly, NY). Customized cRGDyK was purchased from KE Biochem (Shanghai, China). Beta-cyclodextrin (β-CD; MW ~1,134) was purchased from Tokyo Chemical Industry (Tokyo, Japan). Acetic acid, ethyl acetate (EA), Dess-Martin periodinane (DMP), deuterium oxide (D_2O), anhydrous dimethyl sulfoxide (DMSO), sodium acetate, sodium borohydride, sodium hydroxide, and 1-Adamantanecarbonyl chloride was purchased from Sigma-Aldrich (Saint Louis, MO). Ninhydrin agent and succinic anhydride (SA) was purchased from Acros Organics (Morris Plains, NJ). Acetone is from Fisher Scientific (Pittsburgh, PA). All chemicals were used without further purification unless otherwise noted.

The purity of the synthesized compounds was measured using Waters liquid chromatography-mass spectrometry (LC-MS), consisting of an Alliance e2695 separation module, a 2998 PDA detector (212-800 nm), and an Acquity QDA detector (m/z range: 50- 1,239). An XBridge C18 (4.6×150 mm, 5 µm) reverse-phase HPLC column (Waters) was used for LC-MS. The final compounds were purified using Waters preparative HPLC consisting of a 2489 UV/Visible detector, a 1525 Binary HPLC pump, and an XBridge Prep C18 (19×150 mm, 5 µm) reverse-phase HPLC column. The eluent was collected, concentrated by rotary evaporation, and dried under a vacuum overnight.

Preparation of H-dot

Conversion of β-CD to mono-aldehyde β-CD: To synthesize mono aldehyde β-cyclodextrin (Ald-CD), 2 grams of β-cyclodextrin (1.76 mmol) were dissolved in anhydrous dimethyl sulfoxide (DMSO, 20 mL) in a round-bottom flask equipped with a stir bar. Subsequently, 1.12 g of Dess-Martin periodinane (DMP, 2.64 mmol) was added to this solution, and the mixture was stirred at room temperature for 2 h. After this period, the solution was precipitated by adding 250 mL of acetone and left overnight at room temperature. The resulting precipitate was collected via vacuum filtration, redissolved in a minimal amount of DMSO, and then poured into 250 mL of acetone. The mixture was centrifuged at 3000 rpm for 15 min, and the supernatant was discarded. The dissolution of Ald-CD in deionized water (DIW) and subsequent precipitation in acetone were repeated twice, for three precipitation steps, to eliminate soluble impurities. Following the final precipitation, the precipitate was dissolved in 50 mL of distilled water (DIW) and sonicated for 15 min before undergoing

vacuum filtration. The filtrate was then subjected to lyophilization to remove DIW. After lyophilization, a white solid was recovered as the product (> 90%).

Conjugation of mono-aldehyde β-CD (Ald-β-CD) onto EPL: 1.4 g of Ald-β-CD (1.23 mmol) was dissolved in 50 mL of phosphate buffer (pH 8) followed by the addition of 200 mg of EPL (0.05 mmol). The pH of the mixture was adjusted to pH 9 using a 6 M NaOH solution. After stirring for 24 h, 150 mg of sodium borohydride (3.9 mmol) was added to the reaction mixture to reduce the Schiff base to a secondary amine. The mixture was stirred for an additional 24 h at room temperature. After this time period, dynamic dialysis was carried out in a cellulose membrane with a molecular weight cutoff (MWCO) of 6~8 kDa for 72 h against DIW. The dialyzed solution was frozen at -80ºC and lyophilized to afford 900 mg of a white fluffy solid. Size exclusion chromatography was conducted to determine the purity of the CDPL and to confirm the absence of un-grafted Ald-CD. To characterize the β-CD-conjugated EPL (CDPL), the number of β-CD grafted to EPL was determined using ^{1}H-NMR spectroscopy.

Conjugation of ZW800-NHS ester onto CDPL: A ZW800-NHS ester solution was prepared by dissolving 22.94 mg of the dye (22.38 µmol) in 1 mL of dimethyl sulfoxide (DMSO). In a round-bottom flask equipped with a stir bar, a CDPL solution was prepared by dissolving 250 mg of CDPL (18.65 µmol, molecular weight 13,403) in 25 mL of phosphate-buffered saline (PBS) adjusted to pH 9.0. With vigorous stirring, the ZW800-NHS ester solution was added dropwise into the CDPL solution. The reaction mixture was stirred for 3 h while maintaining the pH at 8.0 by adding 0.6 M aqueous sodium hydroxide as necessary. After 3 h, the product was precipitated by adding 250 mL of a 20% ethyl acetate (EA) solution in acetone (%v/v) and then centrifuged at 3000 rpm for 15 min. The supernatant was discarded, and the dissolution of ZW800-CDPL in deionized water (DIW) and precipitation in EA/acetone were repeated twice, totaling three precipitation steps. The resulting product was dried under a vacuum overnight.

Succinylation of ZW800-CDPL: To synthesize zwitterionic ZW800-CDPL, 230 mg of ZW800-CDPL (16.4 µmol) were dissolved in 23 mL of 1x PBS solution (10 mM, pH=7.4) in a round-bottom flask equipped with a stir bar. A 100 µL aliquot of this solution was reserved to serve as a reference for the ninhydrin test later. Succinic anhydride (SA) (31 mg, 310 µmol) dissolved in 125 µL of dimethyl sulfoxide (DMSO) was added dropwise to the ZW800-CDPL

solution under vigorous stirring. To adjust the pH to approximately 9, 6 M aqueous sodium hydroxide (NaOH) solution was added, and the reaction mixture was stirred for 75 min. Subsequently, the ninhydrin test was performed to assess the degree of succinylation. The reaction was terminated by precipitating the reaction mixture in 250 mL of a 20% ethyl acetate (EA) solution in acetone (%v/v), followed by centrifugation of the suspended precipitate for 15 min at 3000 RPM.

Characterization of H-dot

Ninhydrin test for estimation of the number of amine groups: The Ninhydrin reagent was prepared by dissolving 0.8 g of ninhydrin in 10 mL of ethanol. One milliliter of this reagent solution was aliquoted into three separate vials. To the first vial, 20 µL of the reference solution (taken before the addition of succinic anhydride, SA) was added. To the second vial, 20 µL of the reaction mixture (sampled 1 hour after adding SA) was added. The third vial received 20 µL of phosphate-buffered saline (PBS) as a negative control. The vials were vortexed and then heated in a boiling water bath for 5 minutes, followed by immersion in an ice bath for 3 minutes to cool. Each solution was subsequently diluted fivefold with deionized water (DIW), and 1 mL of each diluted solution was transferred to a plastic cuvette. Absorbance measurements at 570 nm were taken using a UV/Vis/NIR spectrometer (USB2000, Ocean Optics, Dunedin, FL), with the negative control serving as the blank. The absorbance at 570 nm of the reaction mixture after 30 minutes was found to be approximately half of that of the reference mixture.

Size-exclusion chromatography analysis to determine hydrodynamic diameters: The purity of H-dots was evaluated through size-exclusion chromatography (SEC) using a Waters HPLC system, which included a Waters e2695 separations module and a Waters 2998 PDA detector. Chromatographic separation was achieved with an Xbridge BEH 125Å 3.5 µm SEC column (7.8 × 150 mm, Waters). The mobile phase was 10 mM phosphate-buffered saline (PBS), maintained isocratically for 16 minutes at a flow rate of 0.75 mL/min. Each component in the reaction mixture was identified by its retention time (Rt) and absorbance wavelength. SEC was performed as described above to determine the hydrodynamic diameter (HD) of the H-dots. A calibration curve was established using protein standards with known HDs. A 10 µL injection of a protein standard mixture, including aprotinin (6.5 kDa, 1.96 nm), ribonuclease (13.7 kDa, 3.28 nm), ovalbumin (44 kDa, 6.10 nm), and thyroglobulin (669 kDa, 9.6 nm), was analyzed under identical mobile phase and flow rate conditions. Although uracil

(112 Da) was part of the mixture, it was excluded from the calibration due to its HD being outside the target range. The partition coefficient (K_{av}) for each protein was calculated using the formula $K_{av} = (V_e - V_0) / (V_c - V_0)$, where V_0 is the column void volume, V_c is the geometric column volume, and V_e is the eluent volume. The K_{av} values were plotted against the logarithm of the proteins' HDs, and a sigmoid curve was fitted to the data. The HD of an unknown sample was determined by converting its Rt to K_{av} and substituting this value into the sigmoid curve equation to solve for HD.

^{1}H-NMR analysis: CDPL was dissolved in D_2O to determine the CD conjugation ratio on the EPL chain. After complete dissolution, the solution was transferred to a clean NMR tube. ^{1}H-NMR spectroscopy was performed on the sample, and the following equation was used to calculate the conjugation ratio:

$$\text{Conjugation ratio} = \frac{64 * \text{H1}}{7 * \varepsilon}$$

, where H1 is the integral area of H1 on β-CD positioned at δ5.06 ppm and ε is the integral area of the protons on EPL positioned at δ3.2 ppm.

Stability of H-dot and RGD/H-dot in different media: H-dot and 4RGD/H-dot were dissolved in PBS (pH 5, 6, and 7) and PBS (pH 5, 6, and 7) supplemented with 10% FBS to a concentration of 100 µM. Each sample (1 mL) was placed into microtubes and incubated at 37 °C for 48 h. At predetermined time points, 100 µL aliquots of each sample were taken and centrifuged at 3000 rpm for 10 min. The UV-Vis absorbance spectra of the supernatants were measured using Cytation5. The absorbance at 758 nm was normalized against the initial absorbance for comparative analysis of the samples.

SUPPLEMENTARY FIGURES

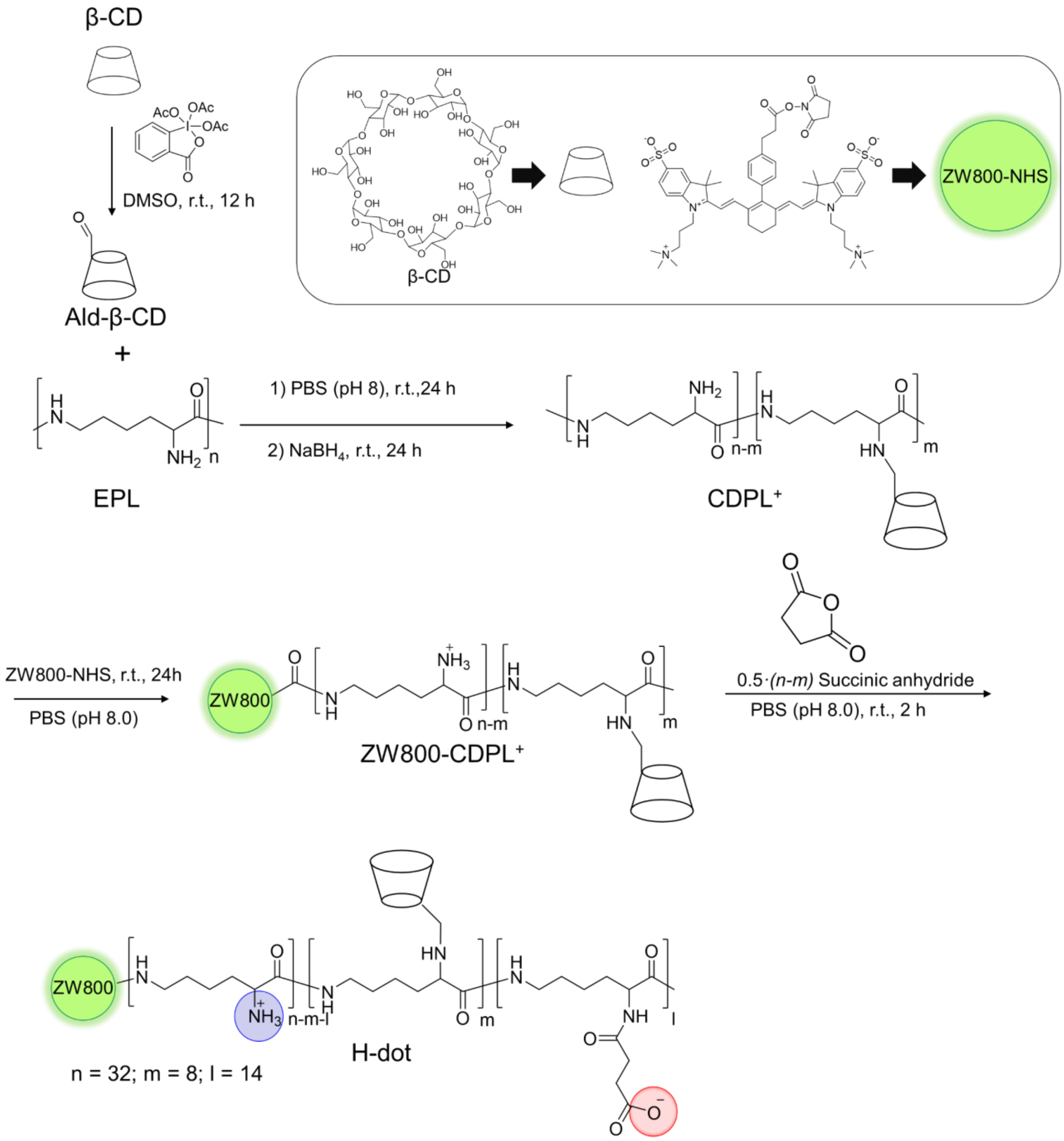

Figure S1. Synthetic procedure for H-dot.

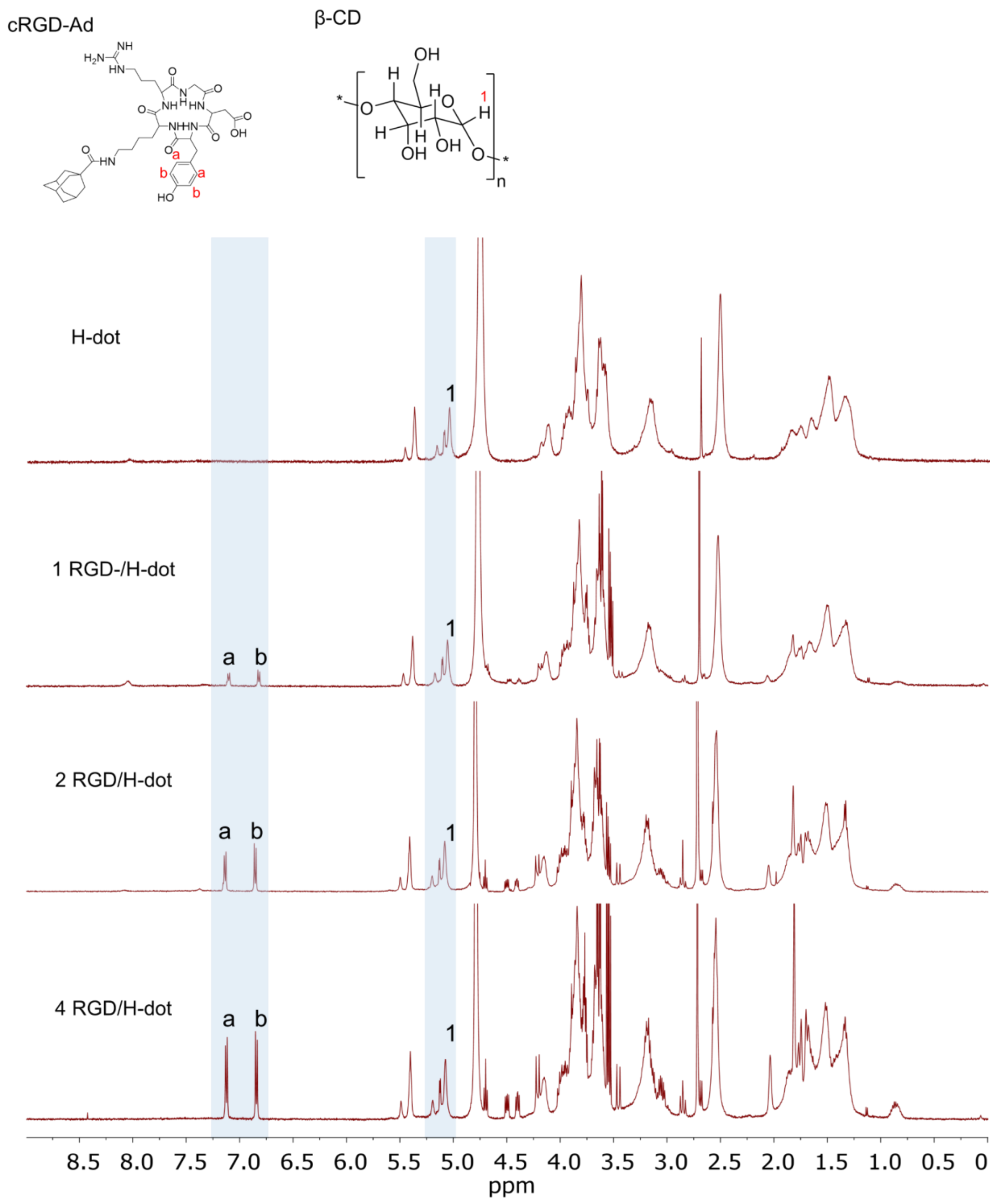

Figure S2. ^{1}H-NMR chromatography of H-dot and RGD/H-dot complexes. The number of cRGD-Ad on H-dot was calculated by peak integration of the β-CD protons (at 5.1 ppm) and cRGD-Ad protons (at 6.8 and 7.1 ppm).

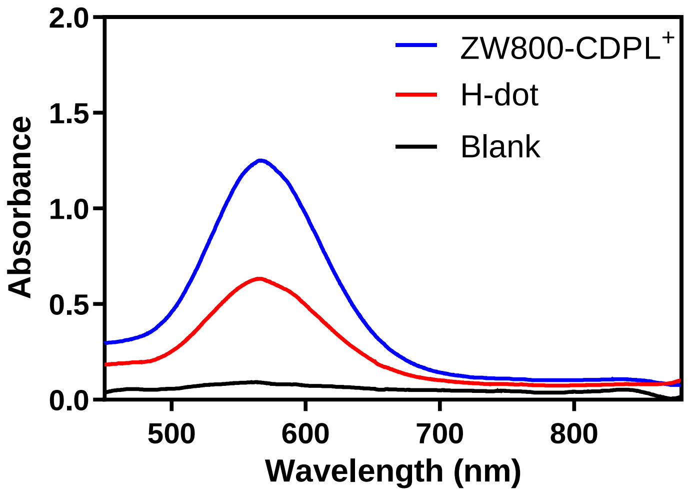

Figure S3. Ninhydrin assay of the amine residue on H-dots. The ninhydrin test was employed to confirm the succinylation ratio of the product molecule. To achieve the zwitterionic property of H-dot, ~50% of succinic anhydrides were applied to the primary amines on ZW800-CDPL^{+}. The absorbance at 570 nm is characteristic of the ninhydrin reagent, indicating the ratio of free primary amines on the H-dot backbone.

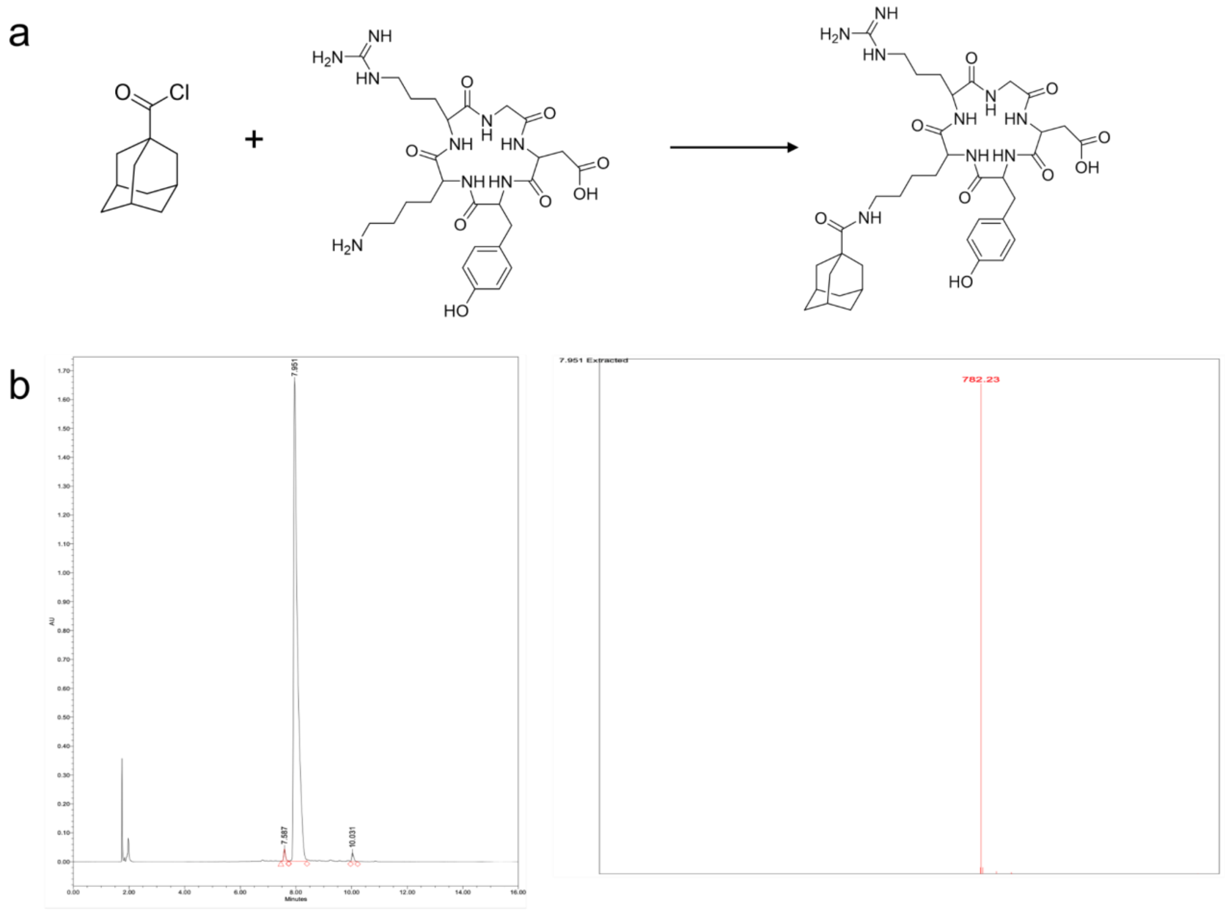

Figure S4. Synthesis of cRGD-Ad. (a) Synthetic route of cRGD-Ad. (b) The HPLC-MS results of cRGD-Ad.

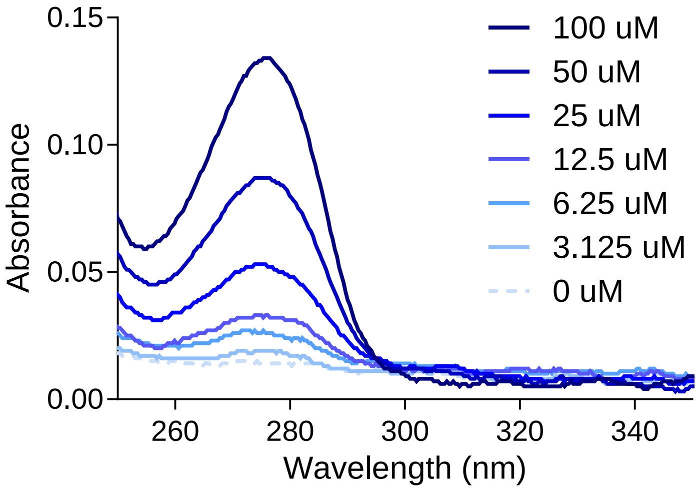

Figure S5. Absorption spectra of cRGD-Ad at various concentrations. The maximum absorbance of cRGD-Ad is found to be at 271 nm.

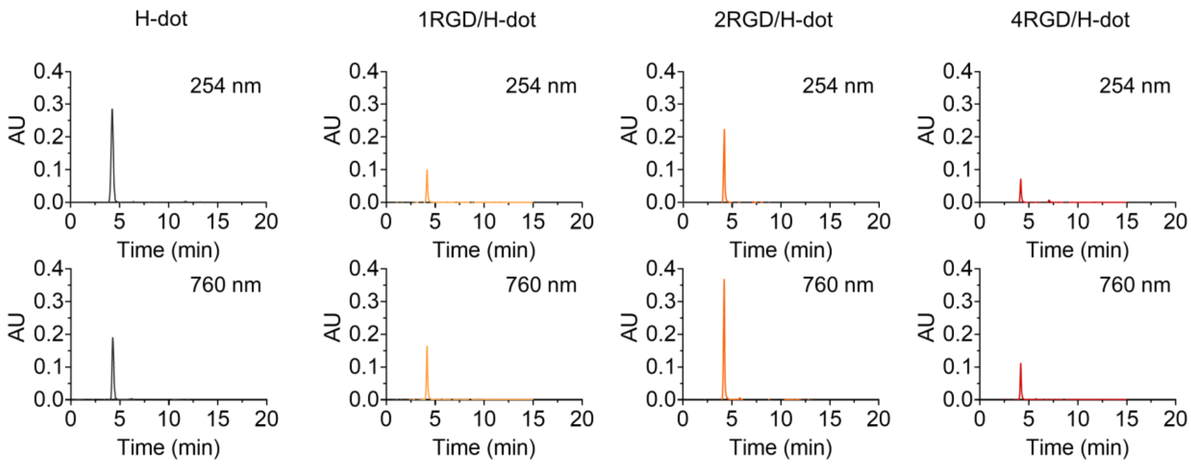

Figure S6. SEC-HPLC spectra for H-dot and RGD/H-dot complexes at 254 and 760 nm channels. The retention times of H-dot and RGD/H-dot complexes were used to calculate the hydrodynamic diameter. A calibration curve was created using standard proteins.

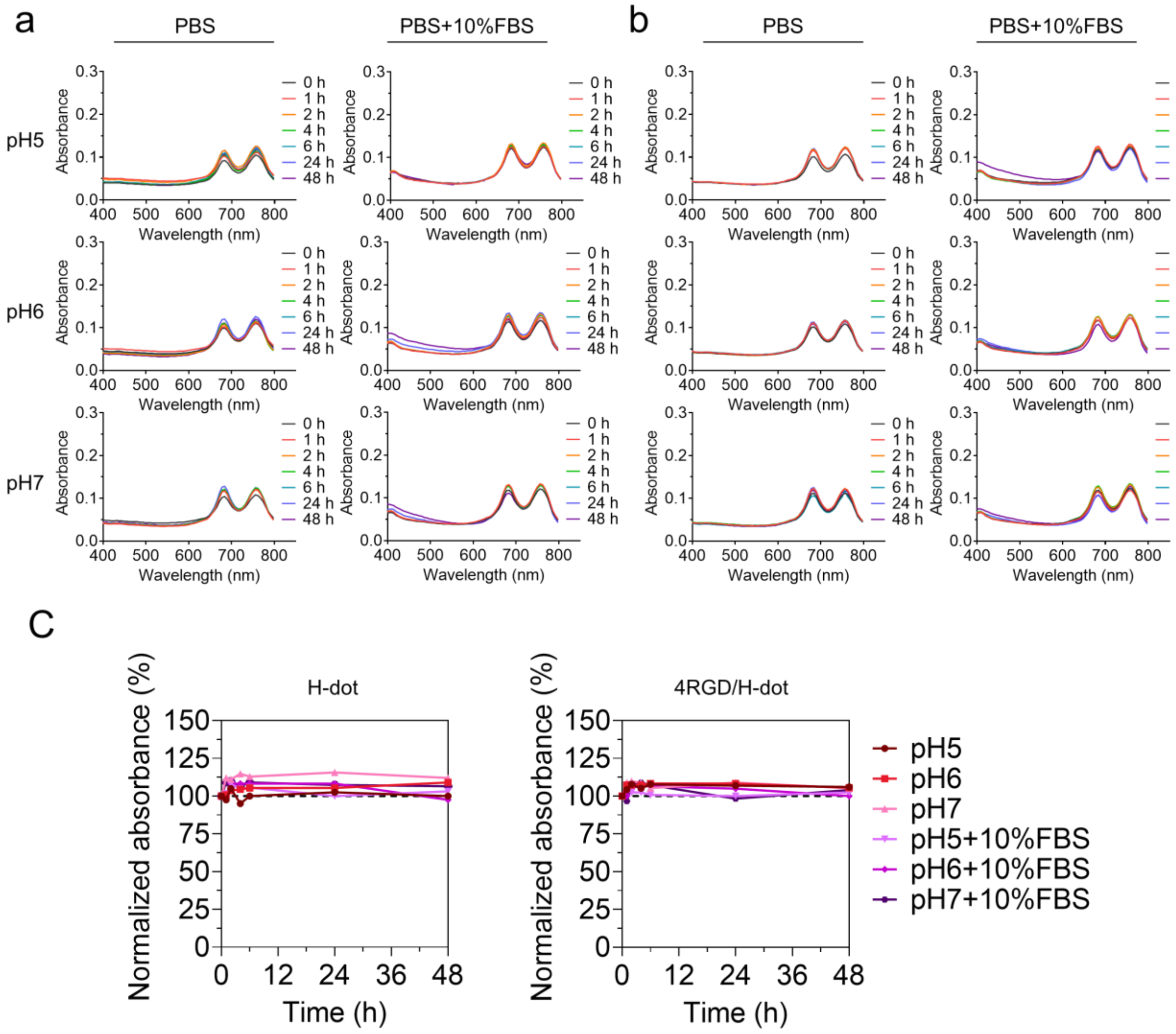

Figure S7. Stability test of H-dot and 4RGD/H-dot in PBS (pH 5, 6, and 7) and PBS (pH 5, 6, and 7) supplemented with 10% FBS. (a) Time-dependent UV-Vis absorbance spectra of H-dot and (b) 4RGD/H-dot. (c) Normalized absorbance at 758 nm of H-dot and 4RGD/H-dot during 48 h.

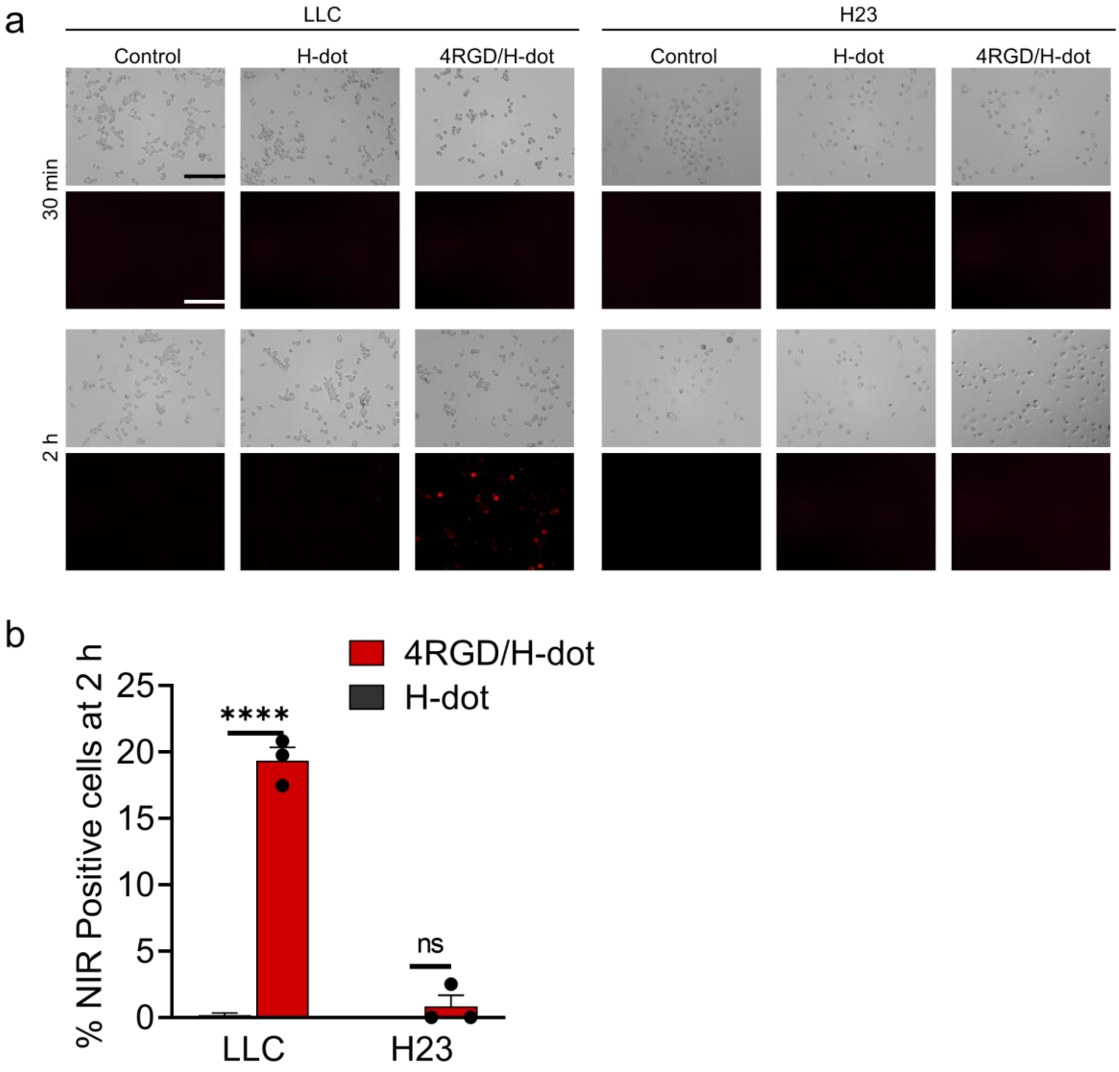

Figure S8. In vitro cellular binding for H-dot and 4RGD/H-dot (5 μM for each) on LLC (left) and H23 cells (right). (a) Bright-field and NIR fluorescence images for LLC and H23 treat with H-dot and 4RGD/H-dot (Scale bar = 100 μm). (b) NIR-positive cells after 2 h. The cells counted in each image are 100-120 and 70-90 for LLC and H23, respectively. ****$p < 0.0001$; n.s. = not significant.

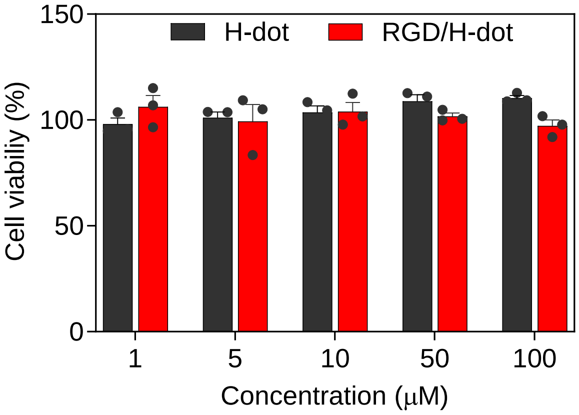

Figure S9. Cell viability of NIH3T3 cell at various concentrations of H-dot and RGD/H-dot. No statistical significance was observed.

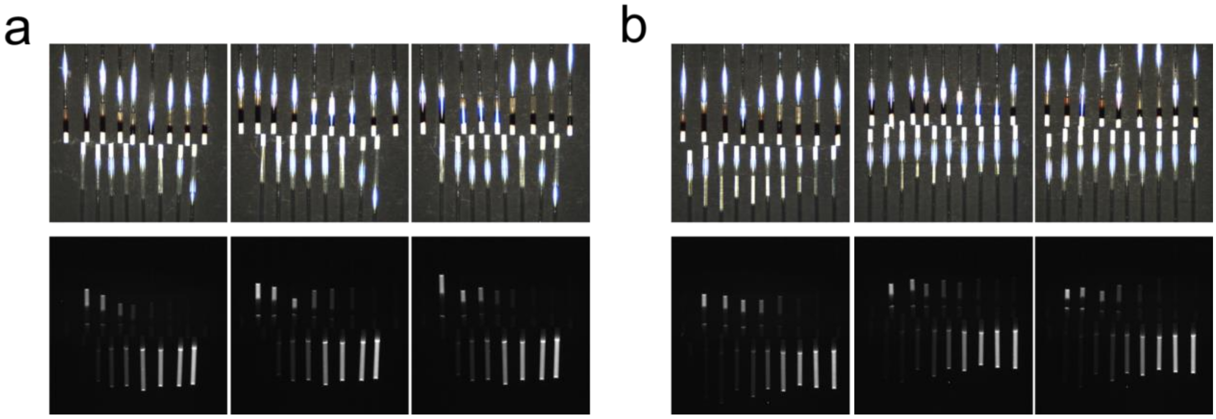

Figure S10. Blood samples in capillary tubes at each time point were used to calculate plasma concentrations of (a) H-dot and (b) 4RGD/H-dot.

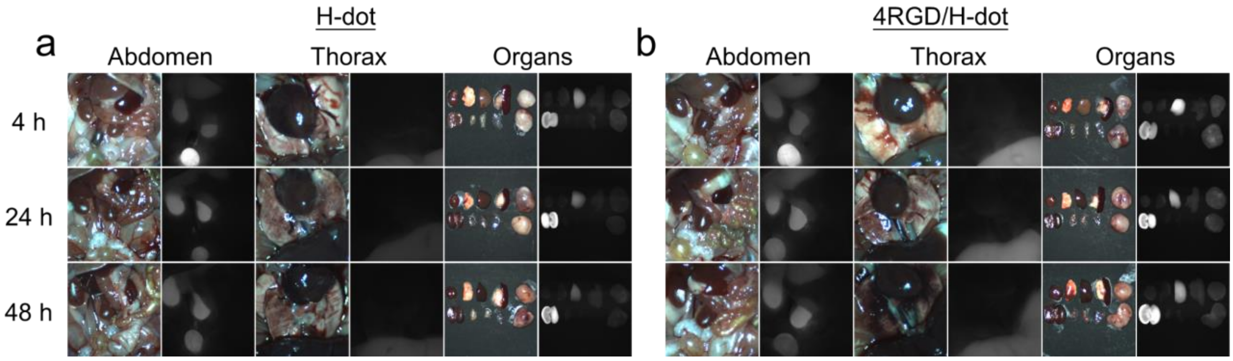

Figure S11. Biodistribution of H-dot and 4RGD/H-dot. (a) Color and NIR fluorescence images of abdomen, thorax, and organs from LLC tumor-bearing mice injected with H-dot and (b) 4RGD/H-dot.
